# Novel computational model of gastrula morphogenesis to identify spatial discriminator genes by self-organizing map (SOM) clustering

**DOI:** 10.1038/s41598-019-49031-1

**Published:** 2019-08-29

**Authors:** Tomoya Mori, Haruka Takaoka, Junko Yamane, Cantas Alev, Wataru Fujibuchi

**Affiliations:** 1 Center for iPS Cell Research and Application (CiRA), Kyoto University, 53 Kawahara-cho, Shogoin, Sakyo-ku, Kyoto 606-8507 Japan; 2 Department of Life Science and Informatics, Faculty of Engineering, Maebashi Institute of Technology, 460-1 Kamisadori, Maebashi City, Gunma 371-0816 Japan; 3 Present Address: Bioinformatics Center, Institute for Chemical Research, Kyoto University, Gokasho, Uji, Kyoto 611-0011 Japan

**Keywords:** Computational models, Embryonic induction, Regenerative medicine, Reverse engineering

## Abstract

Deciphering the key mechanisms of morphogenesis during embryonic development is crucial to understanding the guiding principles of the body plan and promote applications in biomedical research fields. Although several computational tissue reconstruction methods using cellular gene expression data have been proposed, those methods are insufficient with regard to arranging cells in their correct positions in tissues or organs unless spatial information is explicitly provided. Here, we report SPRESSO, a new *in silico* three-dimensional (3D) tissue reconstruction method using stochastic self-organizing map (stochastic-SOM) clustering, to estimate the spatial domains of cells in tissues or organs from only their gene expression profiles. With only five gene sets defined by Gene Ontology (GO), we successfully demonstrated the reconstruction of a four-domain structure of mid-gastrula mouse embryo (E7.0) with high reproducibility (success rate = 99%). Interestingly, the five GOs contain 20 genes, most of which are related to differentiation and morphogenesis, such as activin A receptor and *Wnt* family member genes. Further analysis indicated that *Id2* is the most influential gene contributing to the reconstruction. SPRESSO may provide novel and better insights on the mechanisms of 3D structure formation of living tissues via informative genes playing a role as spatial discriminators.

## Introduction

The reconstruction of three-dimensional (3D) tissues such as organoids and organ-like structures from human induced pluripotent stem (iPS) cells^1^ is one of the most exciting technologies in the field of regenerative medicine. Other techniques, such as cell sheets that can be generated by 3D bio-printers, have been developed, and their usefulness has been reported^2–5^. In recent years, technologies capable of observing the state of cells at single-cell resolution have been developed^6–10^, enabling us to capture the cellular heterogeneity within organs and tissues and identify known and novel subtypes of individual cells^11–14^. Particularly, with the launch of the Human Cell Atlas^15^, a worldwide project aiming to catalog all 37 trillion cells in the human body at the single-cell level, single-cell data production is expected to be accelerated on an unprecedented scale in the near future.

In recent years, several computational methods to reconstruct 3D tissues by estimating the spatial positions of individual cells in tissues with gene expression data obtained by single-cell RNA-seq have been reported^16–22^. These methods may be roughly divided into two types: the landmark approach and the *ab initio* approach. The landmark approach estimates the 3D position of each cell based on gene expression profiles while using the spatial information of marker genes obtained by *in situ* hybridization^16–18^. Conversely, the *ab initio* approach assigns each cell to 3D space according to the principal component score calculated from gene expression profiles without using such spatial reference data^19–22^. Notably, the landmark approach cannot reconstruct tissue structures from *de novo* gene expression profiles without marker genes that provide spatial information. Thus, although current principal component analysis (PCA)-based methods are used for 3D visualization, an *ab initio* approach that does not depend on the spatial information of marker genes obtained by *in situ* hybridization is promising for 3D reconstruction. Previously, we reported a 3D reconstruction method for mouse blastocyst consisting of two cell types that successfully enhances spatial prediction by combining PCA and cell type-specific marker genes coding for cell adhesion molecules^23,24^.

In this study, in order to expand the capability of our preliminary research, we further developed a novel 3D reconstruction method using stochastic self-organizing map (stochastic-SOM) clustering, or SPRESSO (SPatial REconstruction by Stochastic-SOM), which features gene selections based on Gene Ontology (GO)^25,26^. We applied the method to publicly available gene expression data of mid-gastrula mouse embryo (E7.0) to reproduce the embryo’s four domain structure^27^. The method yielded high success rates and demonstrated a remarkable ability to find spatial discriminator genes that contribute to differentiation and tissue morphogenesis.

## Results

### Domain structure of mid-gastrula mouse embryo and RNA-seq samples

**Figure 1:**
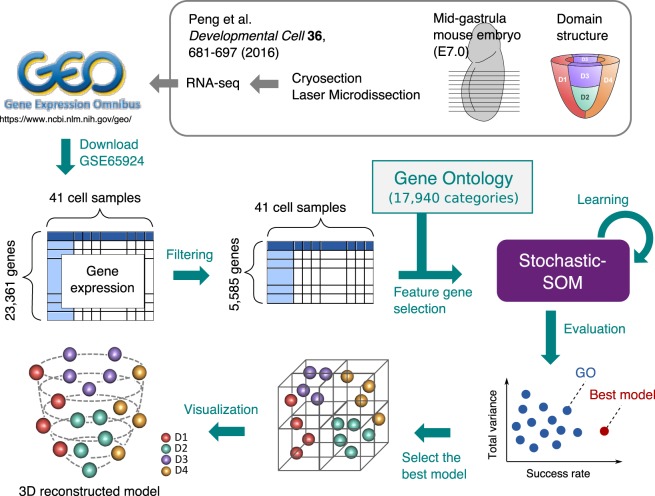
Overview of the 3D reconstruction method of mid-gastrula mouse embryo using stochastic-SOM clustering. The gene expression data of mid-gastrula mouse embryo published by Peng *et al*.^27^ were downloaded from GEO (accession number: GSE65924) and used as input data for our 3D reconstruction method. The expression data consisted of 41 samples with 23,361 genes. After filtering out low-expression genes, we used 5,585 genes as the input data. We generated candidate spatial discriminator gene sets according to GOs. We evaluated all the reconstructed structures from stochastic-SOM clustering in terms of success rate and total variance. Finally, we projected the samples to the paraboloid to reproduce the embryo structure.

To reconstruct the spatial structure of mid-gastrula mouse embryo (E7.0), we used the gene expression profiles in the cryosectioned embryo laser microdissection study reported by Peng *et al*.^27^ (GSE65924) downloaded from GEO (Gene Expression Omnibus)^28^ (Fig. 1). The gene expression profiles for each of the eleven frozen sections were separated into four regions (anterior, posterior, left, and right), for which a total of 41 samples are available (the most distal section has no right or left samples, and there is one low-expression sample). Each sample is composed of a small number of cells (approximately 20 cells per sample) and no single-cell data. The read counts of 23,361 genes by RNA-seq of the 41 samples were normalized by FPKM (fragments per kilobase of transcript per million mapped reads). Peng *et al*. have already reported that the 41 samples can be grouped into four spatial domains (D1: anterior, D2: lateral-distal, D3: lateral-proximal, and D4: posterior) by hierarchical clustering based on differentially expressed genes (DEGs) and PCA^27^. Thus, our 3D reconstruction problem was formulated by the four-domain prediction of the 41 samples, of which 9, 11, 10, and 11 were attributed to D1, D2, D3, and D4, respectively.

### 3D reconstruction with stochastic-SOM clustering

We estimated the 3D positions of the gene expression samples of mid-gastrula mouse embryo using stochastic-SOM clustering. SOM is an unsupervised learning method proposed by Kohonen, which projects high-dimensional data onto a limited number of output classes or units, so that the units with similar sample vectors are located close together on a mapping layer that mimics the brain cortex^29^. As far as we know, there is no method that applies SOM to *in silico* 3D reconstruction based on gene expression data. Although the output layer of SOM is often a two-dimensional (2D) plane, we used a 3D cube composed of two units in each of the three axes (eight units in total) as an output layer in order to reproduce the domain structure of the embryo (Figs 1 and S1). However, because the number of units on the mapping layer was extremely small in our model, the learning often converged to local minima in early steps, and domain separation of the gene samples often became incomplete. Thus, we used the newly developed stochastic-SOM clustering, which gradually converges by introducing a random variable to its neighborhood function (see Methods). The results indicate that stochastic-SOM clustering dramatically improves the balance of divergence and convergence of learning, which is called the cooling schedule in other combinatorial optimization methods such as simulated annealing (Supplementary Fig. S2).

In our 3D reconstruction evaluation, we calculated the success rate and the variance of the reconstruction results. The success rate indicates topological reproducibility and is defined by how frequently the gravity centers of the clustered samples derived from the four domains correctly reproduce topological relationships by 100 iterations with different initial coordinates of the samples. Variance is defined by the sample variance of the 3D coordinates of the clustered samples around the gravity center of each domain and indicates the clustering precision. We determined the average of the variances of the four domains to calculate the total variance.

### 3D reconstruction by PCA and GO-based gene sets

**Figure 2:**
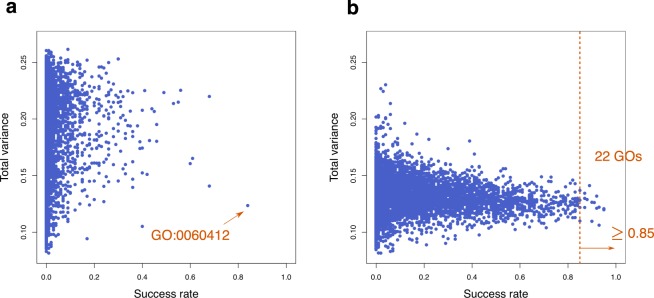
Success rate and total variance of GOs and their combinations. The horizontal and vertical axes show the success rate and total variance, respectively. Each dot indicates a feature gene set selected by GO. (**a**) 6,778 GOs were selected from 17,940 GOs to which the mouse genes belong according to the following two criteria: (i) the number of included genes is less than or equal to 1,000, and (ii) three or more genes from 5,585 genes are contained. GO:0060412 (ventricular septum morphogenesis) shows the highest success rate, 84%. (**b**) The results of all pairs of GO:0060412 and the other 6,777 GOs. The success rates of 22 pairs are equal to or higher than 85%, and the highest is 95%.

After filtering 23,361 genes, Peng *et al*.^27^ performed a clustering of samples based on 158 genes with the top or bottom loading values in the first and second principal components calculated by PCA. Thus, we first performed 3D reconstruction using these 158 genes. Unexpectedly, however, the success rate of the domain topology was only 1%. This result indicates that the genes selected by the PCA are insufficient to properly reproduce the topological relationships of the four domains. We also performed 3D reconstruction analysis using the 1,887 differentially expressed genes reported by Peng *et al*. and the above 23,361 entire gene set, but the success rates were 0% in both cases. Therefore, in order to find the effective gene sets for 3D reconstruction, a comprehensive reconstruction experiment was performed using 17,940 GO gene sets, and the success rate and the total variance were computed for each gene set (Fig. 1). We selected mouse-specific 6,778 GOs (October 17, 2018) with appropriate gene size (1,000 genes or less, and at least three mouse genes after low-expression filtering) out of the 17,940 GOs to exclude too large or too small GOs. Among the 6,778 GOs tested, GO:0060412 (ventricular septum morphogenesis) showed the highest success rate, 84% (Figs 2a and S3a).

### 3D reconstruction by combinations of GO gene sets

To increase the success rate, we further tested the reconstructions by combining all pairs of GO:0060412 and each of the other 6,777 GOs. As a result, 22 pairs exceeded the 84% success rate of GO:0060412, with the highest being 95% (Table 1, Figs 2b, and S3c). We further tested all combinations of GO:0060412 with two other GOs selected from the above 22 GOs (_22_*C*_2_ = 231 combinations) and found five combinations that had success rates exceeding 95%, the highest being 97% (Supplementary Table S1). Furthermore, when we reconstructed four or five GO combinations of GO:0060412 with the other 22 GOs (_22_*C*_3_ = 1,540 and _22_*C*_4_ = 7,315 combinations, respectively), the highest success rate of 99% was observed for five GOs {GO:0060412, GO:0005021, GO:2000392 (or GO:2000394), GO:0031994, GO:0070986} (Supplementary Tables S2 and S3), where the gene sets of GO:2000392 and GO:2000394 were identical. Surprisingly, although the proposed method is based on unsupervised clustering, the GOs related to morphogenesis, such as ventricular septum morphogenesis (GO:0060412), regulation of lamellipodium morphogenesis (GO:2000392 or GO:2000394), and left/right axis specification (GO:0070986), were included to reproduce the 99% success rate. This result suggests that functional gene sets contributing to 3D tissue organization may enhance the frequencies of the reconstructions of the desired tissues. Note that there were no combinations of six GOs (_22_*C*_5_ = 26,334) that exceeded the 99% success rate (Supplementary Table S4). The total number of genes found in the minimum five GOs with the highest success rate (99%) was 20, and many of them were recognized as candidate genes involved in morphogenesis and differentiation, such as activin A receptor, *Wnt*, frizzled, Notch ligand, and so on (Supplementary Table S5). We further tested whether we can increase the success rate by removing genes one by one or combinations from the minimum five GO gene set. We eventually attained 100% success rate by removing two genes, *Arl13b* and *Smad7* (Supplementary Table S6). The final set of 18 genes to reproduce the 100% success rate is shown in Table 2.

**Table 1:** 22 GOs showing success rates of reconstruction equal to or higher than 85% when combined with GO:0060412.

Success rate (%)	Total variance	GO	Term
84 (single GO)	0.124	GO:0060412	ventricular septum morphogenesis
85	0.110	GO:0005021	vascular endothelial growth factor-activated receptor activity
85	0.122	GO:1905456	regulation of lymphoid progenitor cell differentiation
85	0.128	GO:0031117	positive regulation of microtubule depolymerization
85	0.137	GO:0005381	iron ion transmembrane transporter activity
87	0.122	GO:0070986	left/right axis specification
87	0.130	GO:0044117	growth of symbiont in host
87	0.130	GO:0044130	negative regulation of growth of symbiont in host
87	0.130	GO:0044146	negative regulation of growth of symbiont involved in interaction with host
87	0.135	GO:0030169	low-density lipoprotein particle binding
88	0.129	GO:0072079	nephron tubule formation
89	0.141	GO:0003214	cardiac left ventricle morphogenesis
90	0.110	GO:2000392	regulation of lamellipodium morphogenesis
90	0.110	GO:2000394	positive regulation of lamellipodium morphogenesis
90	0.127	GO:0002830	positive regulation of type 2 immune response
90	0.127	GO:0045630	positive regulation of T-helper 2 cell differentiation
91	0.126	GO:0010899	regulation of phosphatidylcholine catabolic process
92	0.121	GO:0042827	platelet dense granule
92	0.132	GO:0048681	negative regulation of axon regeneration
93	0.106	GO:0034707	chloride channel complex
93	0.122	GO:1905564	positive regulation of vascular endothelial cell proliferation
95	0.120	GO:0046716	muscle cell cellular homeostasis
95	0.121	GO:0031994	insulin-like growth factor I binding

**Table 2:** Final set of 18 genes derived from the five GOs reproducing 100% success rate.

Gene	Official full name
*Acvr1*	activin A receptor, type 1
*Cited2*	Cbp/p300-interacting transactivator, with Glu/Asp-rich carboxy-terminal domain, 2
*Coro1b*	coronin, actin binding protein 1B
*Dll1*	delta like canonical Notch ligand 1
*Enpp2*	ectonucleotide pyrophosphatase/phosphodiesterase 2
*Fgfrl1*	fibroblast growth factor receptor-like 1
*Flt1*	FMS-like tyrosine kinase 1
*Fzd1*	frizzled class receptor 1
*Hes1*	hes family bHLH transcription factor 1
*Id2*	inhibitor of DNA binding 2
*Igfbp3*	insulin-like growth factor binding protein 3
*Igfbp4*	insulin-like growth factor binding protein 4
*Itga6*	integrin alpha 6
*Nrp2*	neuropilin 2
*Pdgfra*	platelet derived growth factor receptor, alpha polypeptide
*Rreb1*	ras responsive element binding protein 1
*Slit3*	slit guidance ligand 3
*Wnt5a*	wingless-type MMTV integration site family, member 5A

### Visual inspection of 3D reconstruction and correlation heat map of domain gene expressions

**Figure 3:**
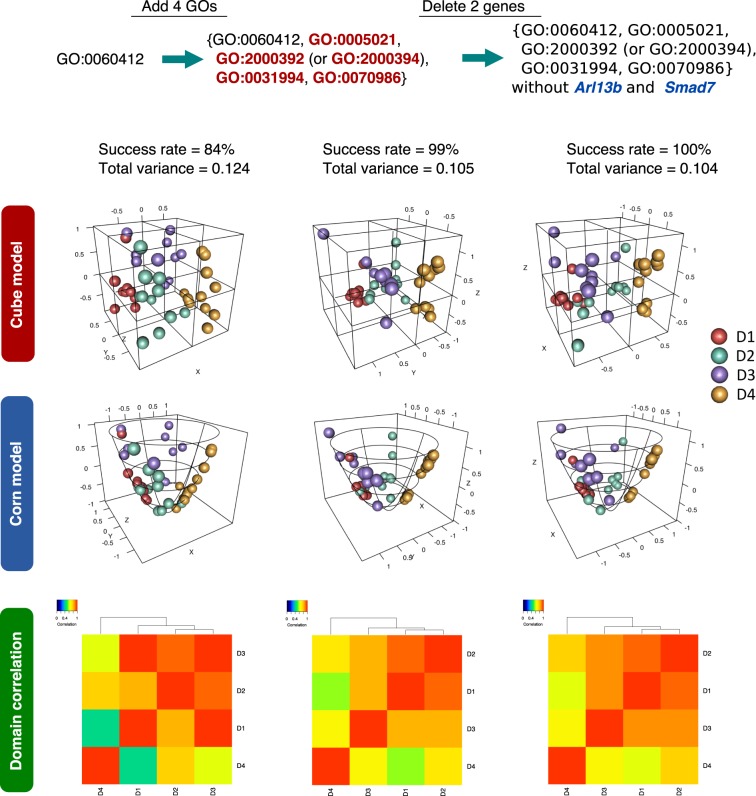
Visualization of reconstructed models from gene expression profiles of mouse embryo samples. Reconstructed mouse embryo models and heatmaps of the domain correlation for different gene sets are shown. When only the feature gene set, GO:0060412, was used, the success rate was 84%. However, when four optimal GOs were added and *Arl13b* and *Smad7* genes were removed, the success rates increased to 99% and 100%, respectively, and the total variances became smaller than that of GO:0060412 only. The visualization distance from the centroids of the output units to each sample reflects the similarity (Euclidean norm) between the centroids and the sample vectors. In the domain correlations, D4 shows a distinct gene expression pattern from the other three domains.

When we inspected the final clusters of samples by our similarity-based visualization method, we found samples from D1 and D4 in diagonal locations, and those from D2 and D3 in up-and-down relationships, consistent with the actual domain structure (Fig. 3). Further, when we inspected domain correlations based on the gravity centers of the gene expressions in the four domains, we found that D1, D2, and D3 were closely clustered, and only D4 showed a gene expression pattern different from the other three domains.

## Discussion

In this study, we developed SPRESSO, an *ab initio* 3D reconstruction method based on stochastic-SOM clustering, to arrange individual samples in a 2 × 2 × 2 cubic structure using their gene expression profiles without any landmark genes. In the computational experiment where we applied our method to the gene expression data of mid-gastrula mouse embryo (E7.0), the embryo’s domain structure was reproduced with a 99% success rate when spatial discriminator gene sets were selected by combinatorial GO optimizations, but not by PCA. Surprisingly, when further optimization by gene deletion was carried out, the total number of genes found in the minimum five GOs with 100% success rate was 18, and many of them were recognized as differentiation- and morphogenesis-related genes (Table 2).

To investigate which genes are the most influential spatial discriminators, we removed each of the 18 genes with 100% success rate to see the reductions of the success rate (Supplementary Table S7). Interestingly, when *Id2* (inhibitor of DNA binding 2) was removed, the success rate was dramatically reduced to 37%. Even when *Id2* was replaced with *Cxcl12*, which has the highest Pearson correlation coefficient (=0.698) with *Id2*, the success rate remained low at 39%. According to the Web page of Jackson Laboratory, *Id2* knockout mice (*Id2*^*tm1Mias*^) exhibit phenotypic changes including “reduced body weight” and “a high degree of homozygous lethality” (https://www.jax.org/strain/028954), indicating that *Id2* is a vital gene. Furthermore, when *Id2* was removed with the other 17 genes as pairs, the success rate was further decreased in 12 of the 17 genes (Supplementary Table S8). The most influential gene pair was *Id2* with *Nrp2* (neuropilin 2), which yielded only 7% success rate when removed. Thus, *Id2* and *Nrp2* may be considered strong spatial discriminator genes that contribute to the arrangement of the 3D positions of samples in the mid-gastrula embryo structure. ID proteins function as positive regulators of cell proliferation and negative regulators of cell differentiation^30^. According to iTranscriptome^27,31^ (http://www.picb.ac.cn/hanlab/itranscriptome), there is a gene expression gradient of *Id2* from the anterior proximal side to the posterior distal side of the mid-gastrula embryo (Supplementary Fig. S4). Therefore, we conclude that *Id2* may provide strong spatial information for both the anterior-posterior and proximal-distal axes, which leads to a 63% reduction of the success rate even by the removal of a single gene. The *Nrp2* gene encodes a transmembrane receptor protein that binds to secreted semaphorin 3C and 3F proteins and interacts with vascular endothelial growth factor (VEGF). The *Nrp2* gene is involved in early embryonic and cardiovascular development, axon guidance, and tumorigenesis^32–35^. The gene expression gradient pattern of *Nrp2* is very similar to that of *Id2*; *Nrp2* shows high expression in the anterior proximal region. However, *Nrp2* also shows relatively higher expression levels than *Id2* in the posterior proximal region, and the expressed samples are mostly observed in the proximal region (Supplementary Fig. S4).

When we scrutinized other genes in the best 18 gene set to determine which ones decrease the success rate most when removed with *Id2*, we found that the top five genes were *Nrp2*, *Fzd1*, *Hes1*, *Enpp2*, and *Acvr1* in this order (Supplementary Table S8). *Fzd1* is a member of frizzled gene family encoding 7-transmembrane domain proteins, which act as receptors for WNT signaling proteins and are involved in embryonic development^36,37^. HES1 (hairy and enhancer of split-1) is a bHLH transcription factor involved in cell proliferation and differentiation during embryogenesis^38–40^. *Enpp2* (ectonucleotide pyrophosphatase/phosphodiesterase 2) is reported in the context of neural development, and its deletion leads to embryonic lethality at an early stage^41,42^. ACVR1 (activin A receptor, type I) protein is part of the bone morphogenic protein (BMP) pathway and involved in the development and repair of the skeletal system^43–45^. ACVR1 is also reported in the context of gastrulation and expressed along the entire axis of the primitive streak^46^. Expectedly, all of the top five genes are involved in development, differentiation, cell proliferation, or morphogenesis. According to iTranscriptome, the expressions of all five genes are biased to some regions, such as proximal, anterior, posterior, or combinations of these three, indicating that they are informative genes that play the role of spatial discriminators.

In the SPRESSO analysis, an exhaustive search of all combinations of genes up to some particular gene set size, such as 18, is too computationally expensive (we estimate ~10^43^ years on our cluster machine with 296 cores). Therefore, we performed a limited but promising approach to produce suboptimal combinations of genes through GOs. To confirm the validity of our method, we performed the following two computational tests: (i) First, we generated 6,778 groups of genes with the same size distribution of the 6,778 GOs by random shuffling the gene pairs one million times, and then ran the stochastic-SOM clustering and performed the same series of analysis. Interestingly, the highest success rate was 89% with the best 2 GOs, leaving as few as six genes (*Egr1*, *Junb*, *Lag3*, *M1ap*, *Nefl*, and *Sirt7*), and the success rate did not increase after removing genes. When we scrutinized the total variances, we found drastic changes, i.e., the distribution of the total variances significantly shifted to higher values, and the total variance by the above six genes was 0.164 while that of the original data was only 0.10 (Figs 3 and S3b,d). Thus, GOs are meaningful for selecting gene sets that give not only high success rates but also low total variances, while randomized GO data may happen to give high success rates but are inferior in total variances to the original GO data. (ii) Second, we searched for a smaller gene set that can produce a high success rate by starting with each of all 5,585 genes contained in the 6,778 GOs and adding genes one by one to the gene set of the highest success rates until we found a gene set of 100% success rate. As a result, 10 combinations of four genes (*Tnfrsf1a*, *Nefl*, *Myb*, and one of *2210016L21Rik*, *2900055J20Rik*, *AA465934*, *Gm19710*, *Htra3*, *Katna1*, *Pan2*, *Siah1a*, *Tnnc2*, or *Uba1*) showed 100% success rates. However, these gene sets, similar to the gene-randomized GO test, showed total variances as high as 0.18–0.20, indicating that the direct gene combinatorial method with smaller gene sets tends to find local optimal solutions with higher variances. It should be noted that these genes have no strong relationships with early embryonic development or morphogenesis.

Another major concern of our method is whether it can reproduce similar results against noise. To investigate the robustness and sensitivity to noise, we added Gaussian noise to each of the best 18 genes with the same standard deviation by 1–10% of the whole data and examined the 3D structure reproducibility by SPRESSO (Supplementary Fig. S5). As a result, the data with 1%, 2%, and 5% noise levels produced success rates as high as 94.9%, 91.3%, and 80.3%, respectively, with total variances as low as 0.10–0.11. However, the data with 10% noise produced only 65.8% success rate, with a higher total variance of 0.12. Thus, we conclude that our method is robust and reproducible with conventional noise levels, such as <5%, in the data. Furthermore, we investigated GO gene set size dependencies (Supplementary Fig. S3e–h). The results indicate that the gene size of the GOs is not an important factor for a higher success rate, but the signal-to-noise ratio (SNR) might be. In other words, as the total number of genes increases, the fraction of non-spatial discriminator genes in the gene set may also increase, which will cause noise in the SOM clustering process and thus reduce the success rate.

Our method is intended not only to find influential spatial discriminator genes but also to computationally reproduce tissue 3D structures from a large amount of single-cell transcriptome data, in which positional information in the original tissue structure was lost in the single-cell analysis. To effectively reproduce the original tissue structure, however, one first needs to identify spatial discriminator gene sets, which may be unique to each tissue type, with at least one learning data set consisting of gene expression data and domain positions of cells for each tissue type of interest. The positional information will be quite useful to annotate cell types as well as to estimate cellular functions. It should be emphasized that our method is not only an improvement of existing methods, but also a novel *ab initio* 3D tissue reconstruction approach. Self-organization is a well-known principle that has been studied in developmental biology for many years. Although the reasons why stochastic-SOM clustering can contribute to reproducing the 3D domain structures of early mouse embryo remain unknown and are still under investigation, we speculate that some kind of domain-to-domain similarities, such as hierarchical gradients of morphogens or other structure contributor gene expressions, might be important factors that influence the spatial relationships of cell types^47,48^. Further investigations of the spatial discriminator genes obtained by our method are warranted to enhance our understanding of 3D structure organization models based on the coordinated gene expressions of living tissues in the future.

Finally, there is a limitation of this SOM approach; it may not be usable for other distinct or exclusive samples that show totally different gene expression patterns. For those cases, different mechanisms of interactions (e.g., ligand-receptor interactions) must be added to the constraints of the SOM clustering. For more complex structures, it is further necessary to change the implementation of the SOM structure model. We are currently developing an alternative method based on interaction models, which may recapitulate complicated structures more easily and with more domains from various kinds of tissues or organs than the current cubic structure model.

## Methods

### Preparation of candidate spatial discriminator gene sets

To extract spatial discriminator genes containing information crucial for reconstructing a 3D tissue structure from all 23,361 mouse genes, we removed low-expression genes as follows. We initially extracted 5,585 genes with FPKM values greater than 1 in at least two of all 41 samples and variance of log_10_ (FPKM + 1) across the 41 samples greater than 0.05 for the discriminator gene set selection. Regarding the spatial discriminator gene set obtained by PCA by Peng *et al*.^27^, genes with the top and bottom 40 loading values from the first and second principal components, respectively, were used (a total of 158 unique genes). We also selected 6,778 GO gene sets from all 17,940 mouse GOs (October 17, 2018) on the basis of two criteria: (i) the number of included genes is less than or equal to 1,000, and (ii) three or more genes from the aforementioned 5,585 genes are contained. Although the GO gene data set was extracted by the R/Bioconductor package “biomaRt” (Ensembl Release 94), information of the descendant GO genes was not included in the data set. Thus, the “GO.db” package was used to obtain all offspring GO gene sets to build complete GO gene data sets.

### Stochastic self-organizing map (stochastic-SOM) clustering

Before presenting stochastic-SOM clustering, we briefly review the general SOM clustering algorithm, which is based on the unsupervised learning proposed by Kohonen^29^. The general SOM projects high-dimensional data onto a limited number of output classes or units, so that different units with similar centroid vectors are located close together on a mapping layer that mimics the brain cortex. Let the *p*-dimensional sample vectors *j* (*j* = 1, 2, …, *n*) given as input be ***x***_*j*_ = (*x*_*j*1_, *x*_*j*2_, …, *x*_*jp*_). The 2D space of the output layer is composed of *k* units, and weight vector ***m***_*i*_ = (*m*_*i*1_, *m*_*i*2_, …, *m*_*ip*_) (*i* = 1, 2, …, *k*) is allocated to each unit. We initially calculate the Euclidean distance between input sample *j* and all units *i*, and find that unit *c* is the best matching unit (BMU) with the highest similarity, according to Eq. (1):

1 $$c={\rm{\arg }}\mathop{\min }\limits_{i\in \{1,\,\cdots ,\,k\}}\{\Vert {{\boldsymbol{x}}}_{j}-{{\boldsymbol{m}}}_{i}(t)\Vert \},$$

where $\Vert \,\cdot \,\Vert$ indicates the Euclidean distance, or norm of a vector. The weight vector ***m***_*i*_ (*t*) of all units of the output layer at time *t* is updated based on Eqs (2) and (3):

2 $${{\boldsymbol{m}}}_{i}(t+1)={{\boldsymbol{m}}}_{i}(t)+{h}_{ci}(t)({{\boldsymbol{x}}}_{j}-{{\boldsymbol{m}}}_{i}(t))$$

3 $${h}_{ci}(t)=\alpha (t)\exp (-\frac{{\Vert {{\boldsymbol{r}}}_{c}-{{\boldsymbol{r}}}_{i}\Vert }^{2}}{2{\sigma }^{2}(t)}),$$

where *h*_*ci*_(*t*) is called a neighborhood function and is determined by the distance from unit *c* and constrains how much ***m***_*i*_(*t*) receives the learning influence of ***x***_*j*_ when it is being updated. *α*(*t*) and *σ*(*t*) are the learning rate and function, respectively, that define the radius of the neighboring region. ***r***_*c*_ and ***r***_*i*_ are position vectors in the output layers of units *c* and *i*. The SOM algorithm repeats updates of ***m***_*i*_ until learning step *t* reaches the number of learning times *T* given in advance for all input samples *j*. In the general SOM, the result varies depending on the order in which sample *j* is input, so that a batch-learning SOM has been proposed so that the input order does not affect the result. In the batch-learning SOM, each learning step is executed by Eqs (4) and (5):

4 $${c}_{j}(t)={\rm{\arg }}\mathop{\min }\limits_{i\in \{1,\,\cdots ,\,k\}}\{\Vert {{\boldsymbol{x}}}_{j}-{{\boldsymbol{m}}}_{i}(t)\Vert \}$$

5 $${{\boldsymbol{m}}}_{i}(t+1)=\frac{{\sum }_{j=1}^{n}{h}_{{c}_{j}(t)i}(t){{\boldsymbol{x}}}_{j}}{{\sum }_{j=1}^{n}{h}_{{c}_{j}(t)i}(t)}.$$

In the developed method, we implement a 3D batch-learning SOM in which the output layer is extended from 2D to 3D space. The output layer has a structure of a 3D cube composed of a total of eight units in which two units are assigned to each of the *x-*, *y-*, and *z*-axes (Supplementary Fig. S1a). This assumes that the mid-gastrula mouse embryo structure consists of four parts (anterior, posterior, left, and right) on the *xy*-plane and two parts (proximal and distal) on the *z*-axis (Supplementary Figs S1b and S6a). Although the weight vectors of BMUs and their adjacent units are updated according to neighborhood function *h*_*ci*_(*t*) in the normal learning step, we introduce the constraint in which the diagonal units on the *xy*-plane are not updated, because actual mid-gastrula mouse embryo has a hollow structure in the middle of the body and the diagonal units are not spatially connected.

In the general SOM, if the number of units in the output layer is extremely small, the learning often converges to local minima in early steps (Supplementary Fig. S2a). Therefore, by introducing a random variable to neighborhood function *h*_*ci*_(*t*) at time *t*, we achieve stochastic-SOM clustering, which makes the learning process converge gradually (Supplementary Fig. S2b). The neighborhood function of the stochastic-SOM clustering is shown as equation (6):

6 $${h}_{ci}(t)=\alpha (t)\exp (-\frac{{\rm{rnd}}[0.5,\,1)\cdot {\Vert {{\boldsymbol{r}}}_{c}-{{\boldsymbol{r}}}_{i}\Vert }^{2}}{2{\sigma }^{2}(t)}),$$

where rnd [0.5, 1) is a function that generates a uniform random value between 0.5 and less than 1.0. For each set of different input genes, we perform 100 trials of reconstructions starting from different initial weight vectors because the clustering result of SOM is affected by the initial parameters of the map. In this computational experiment, the initial values of neighborhood region *σ* and learning rate *α* of the map are set to 0.6 and 1.0, respectively, and the random seed is changed from 0 to 99. Stochastic-SOM clustering is implemented using programming language Python with “Pandas” and “NumPy” packages for manipulation of the gene expression data and matrix calculation.

### Visualization of 3D reconstructed mid-gastrula mouse embryo structure

We visualized the mid-gastrula mouse embryo structure by projecting samples on a paraboloid based on position information estimated by stochastic-SOM clustering (Fig. 1). Here, we devised a similarity-based visualization that considers similarity between the weight vector of each unit and the sample coordinates as the projecting position (see Supplementary Information).

### Success rate and variance

Evaluation of the reconstructed models was carried out by comparing the topological relationships of the gravity centers of the four domains, D1 to D4, of the reconstructed model with the domain topology of the actual mid-gastrula mouse embryo structure. We first assigned D1 to D4 to units with the SOM clustering results using the gravity centers (*x*_*Di*_, *y*_*Di*_, *z*_*Di*_) (*i* = 1, 2, 3, 4) of samples from the original domains. An evaluation value, *s*, which indicates whether the four domains are correctly arranged or not, was obtained by the following calculations (Steps 1 to 5) (Supplementary Fig. S6):

- (Step 1) *s*′←0
- (Step 2) *s*′←*s*′ + 1 if D1 and D4 are positioned diagonally on the *xy*-plane
- (Step 3) *s*′←*s*′ + 1 if D2 and D3 are adjacent in the *z*-axis
- (Step 4) *s*′←*s*′ + 1 if D*i* (*i* ∈ {1, 4}) and D*j* (*j* ∈ {2, 3}) are adjacent on the *xy*-plane
- (Step 5) *s* ← 1 if *s*′ is equal to _4_*C*_2_, otherwise, *s* ← 0.

That is, *s* becomes 1 if and only if the relative positions of the expression domains are equivalent to that of an actual embryo’s domain structure for all pairs of domains. Through 100 iterations, we compute the “success rate” of each input feature gene by equation (7):

7 $$Success\,rate=\frac{1}{T}\mathop{\sum }\limits_{t=1}^{T}{s}_{t},$$

where *T* is the number of iterations, and *s*_*t*_ is score *s* at the *t*-th iteration.

In addition to the success rate, we introduced another criterion, “total variance,” which is defined by equation (8). Total variance indicates the degree of convergence of the samples for each domain:

8 $$Total\,variance=\frac{1}{T}\cdot \frac{1}{3D}\mathop{\sum }\limits_{t=1}^{T}\mathop{\sum }\limits_{i=1}^{D}({v}_{{t}_{{i}_{x}}}+{v}_{{t}_{{i}_{y}}}+{v}_{{t}_{{i}_{z}}}),$$

where *D* is the number of domains, and ${v}_{{t}_{{i}_{x}}}$, ${v}_{{t}_{{i}_{y}}}$, and ${v}_{{t}_{{i}_{z}}}$ are the unbiased variance of the sample positions of domain *i* at the *t*-th iteration for the *x*-, *y*-, and *z*-axes, respectively.

### Domain correlation

The correlations of the four domains are computed by using the gravity centers of samples belonging to individual domains. The computation is done by the “cor” function of R standard library, and the heatmaps in Fig. 3 are drawn by the “heatmap.2” function of the “gplots” package and “rich.colors” function of “RColorBrewer” on R.

## Supplementary information

Supplementary Information

## Data Availability

The mid-gastrula mouse embryo gene expression data used in this study were published by Peng *et al*.^27^ and are deposited in NCBI GEO under accession number GSE65924 (Supplementary file: GSE65924_E1.gene.expression.txt.gz).

## Code Availability

The proposed methods including the feature gene selection, the 3D reconstruction using stochastic-SOM clustering, and visualization, are implemented in R and Python and are available at http://github.com/tmorikuicr/spresso.
