# Supplementary material for: Novel computational model of gastrula morphogenesis to identify spatial discriminator genes by self-organizing map (SOM) clustering
Source: Sci Rep. 2019 Aug 29;9:12597. doi: 10.1038/s41598-019-49031-1 (PMC6715814; doi:10.1038/s41598-019-49031-1)
Supplement: Supplementary file 1 — Supplementary Information [file 41598_2019_49031_MOESM1_ESM.docx]

**Supplementary information for “Novel computational model of gastrula morphogenesis to identify spatial discriminator genes by self-organizing (SOM) clustering”**

**Authors**

Tomoya Mori^1†^, Haruka Takaoka^2^, Junko Yamane^1^, Cantas Alev^1^, and Wataru Fujibuchi^1,*^

**Affiliations**

^1^ Center for iPS Cell Research and Application (CiRA), Kyoto University, 53 Kawahara-cho, Shogoin, Sakyo-ku, Kyoto 606-8507, Japan

^2^ Department of Life Science and Informatics, Faculty of Engineering, Maebashi Institute of Technology, 460-1 Kamisadori, Maebashi City, Gunma 371-0816, Japan

^*^ Corresponding author

E-mail: [fujibuchi-g@cira.kyoto-u.ac.jp](mailto:fujibuchi-g@cira.kyoto-u.ac.jp)

^†^ Present address: Bioinformatics Center, Institute for Chemical Research, Kyoto University, Gokasho, Uji, Kyoto 611-0011, Japan.

**Supplementary Information**

**Visualization of 3D reconstructed mid-gastrula mouse embryo structure**

We visualized the mid-gastrula mouse embryo structure by projecting samples on a paraboloid based on position information estimated by stochastic-SOM clustering (Fig. 1). Here we propose a similarity-based visualization method. Let the gravity center of eight units of the 3D cube of the SOM output be the overall origin, and the gravity center of each unit $i$ be $(x_{i}, y_{i}, z_{i})$ $(i=1,2,\ldots,8)$, where the values of $x_{i}$, $y_{i}$, and $z_{i}$ are one of eight combinations of either 0.5 or -0.5. As a result of learning, when input sample $j$ is classified into unit $i$, sample $j$ is projected to point $(x_{j}^{'},y_{j}^{'},z_{j}^{'})$ on the paraboloid whose vertex is (0, 0, -1) (Supplementary Fig. S1). The similarity-based visualization considers the similarity between the weight vector of each unit and the sample coordinates as the projecting position through the following steps:

1. Calculate weight vector $\boldsymbol{m}_{i}$ for each output unit $i=\left( x_{i},y_{i},z_{i} \right)$.
2. Let $A$ and $B$ be the centroid vectors of the unit to which sample $j$ belongs and unit $k$ to which sample *j* is adjacent in the $x$-axis, respectively, and then calculate the vector with $A$ as the starting point and $B$ as the ending point.
3. Find the nearest point $X$ on line $AB$ from position $P$ (i.e., the foot of the perpendicular) of sample $j,$ and calculate distance $\left\| \boldsymbol{x} \right\|$ of line segment $AX$:

| $\left\Vert\boldsymbol{x} \right\Vert=\frac{\boldsymbol{b}\cdot\boldsymbol{p}}{\left\Vert\boldsymbol{b} \right\Vert},$ | (S1) |
| --- | --- |

where $\boldsymbol{x}=\vec{AX}$, $\boldsymbol{b}=\vec{AB}$, $\boldsymbol{p}=\vec{AP},$ and $\left\| \cdot\right\|$ indicates the norm of a vector (Supplementary Fig. S7).

1. Calculate $d_{x}$ by normalizing $\left\| \boldsymbol{x} \right\|$ with $\left\| \boldsymbol{b} \right\|$.

| $d_{x}=\frac{\left\Vert\boldsymbol{x} \right\Vert}{\left\Vert\boldsymbol{b} \right\Vert}$ | (S2) |
| --- | --- |

1. For the $y$- and $z$-axes, perform (Step 2) to (Step 4), and calculate $d_{y}$ and $d_{z}$ in the same manner.
2. Define the coordinates of sample $j$ belonging to output unit $i$ as $\left( x_{j}, y_{j}, z_{j} \right)=\left( x_{i}+d_{x}, y_{i}+d_{y}, z_{i}+d_{z} \right)$ (Supplementary Fig. S8).
3. Project samples to a paraboloid by equations (S3), (S4), and (S5).

| $\left( x_{j}^{'}, y_{j}^{'}, z_{j}^{'} \right)=\left( r_{j}\cos\theta,r_{j}\sin\theta, z_{j} \right),$ | (S3) |
| --- | --- |
| $r_{j}=\sqrt{z_{j}+1},$ | (S4) |
| $\theta=\tan^{-1} \frac{y_{j}}{x_{j}} .$ | (S5) |

The visualization program was implemented using the package "rgl" of R language.

**Supplementary Figures**

**Supplementary Figure S1.** Visualization of mid-gastrula mouse embryo structure. (**a**) An output layer of 3D SOM composed of eight units. The black dot indicates input sample $j$ belonging to unit $i.$ (**b**) The paraboloid imitating the structure of mid-gastrula mouse embryo. Input sample $j$ is mapped to point $(x_{j}^{'}, y_{j}^{'},z_{j}^{'})$ on the paraboloid.

**Supplementary Figure S2.** Convergence curves of SOM and stochastic-SOM. The panels show the convergence curves when changing the seed values that determine the initial states of (**a**) SOM and (**b**) stochastic-SOM using GO:0060412 as the feature gene set. The horizontal and vertical axes show, respectively, the learning steps and the number of samples moved to different units from the previous steps by learning. The curves of stochastic-SOM converge more gradually than those of a standard SOM.

**Supplementary Figure S3.** Success rate, total variance, number of genes for GOs, and random gene sets and their combinations. Success rates and total variances are shown for (**a**) 6,778 GOs, (**b**) randomized gene set GOs by shuffling gene pairs in all 6,778 GOs, (**c**) pairs of GO:0060412 and each of the other 6,778 GOs, and (**d**) pairs of the best random gene set in (**b**) and each of the other 6,777 random gene sets. The total variances of randomized GO pairs in (**d**) are significantly shifted to higher values compared with the raw GO pairs in (**c**). Plots of success rates against the gene sizes are shown in (**e**), (**f**), (**g**), and (**h**) for the datasets of (**a**), (**b**), (**c**), and (**d**), respectively. The upper right sub-panels in (**e**), (**f**), (**g**), and (**h**) are close-ups for 0 to 50 genes. Gene sets of moderate size (25 or less) tend to show higher success rates, although some larger gene sizes, such as 50 or more, show higher success rates too.


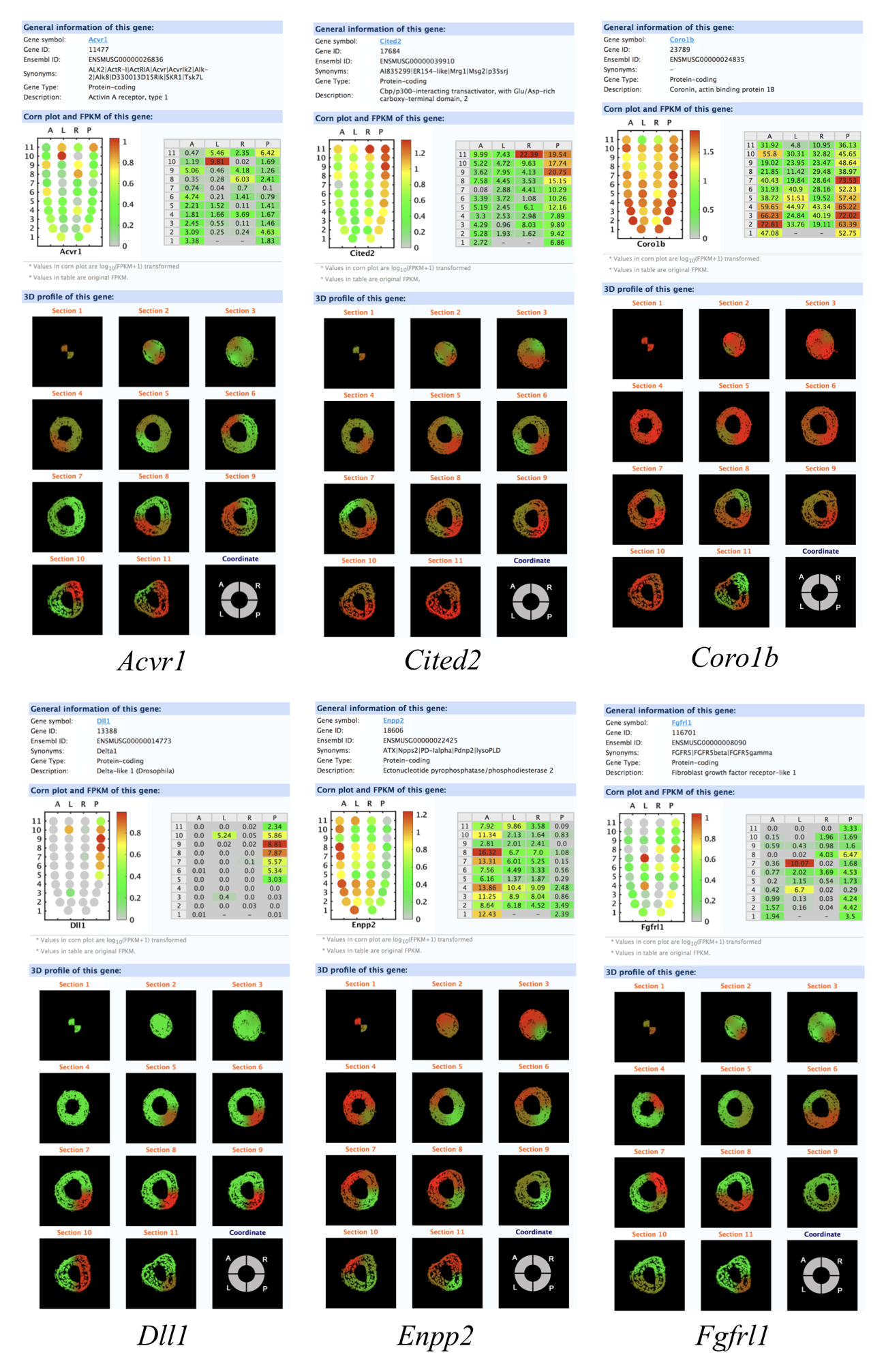


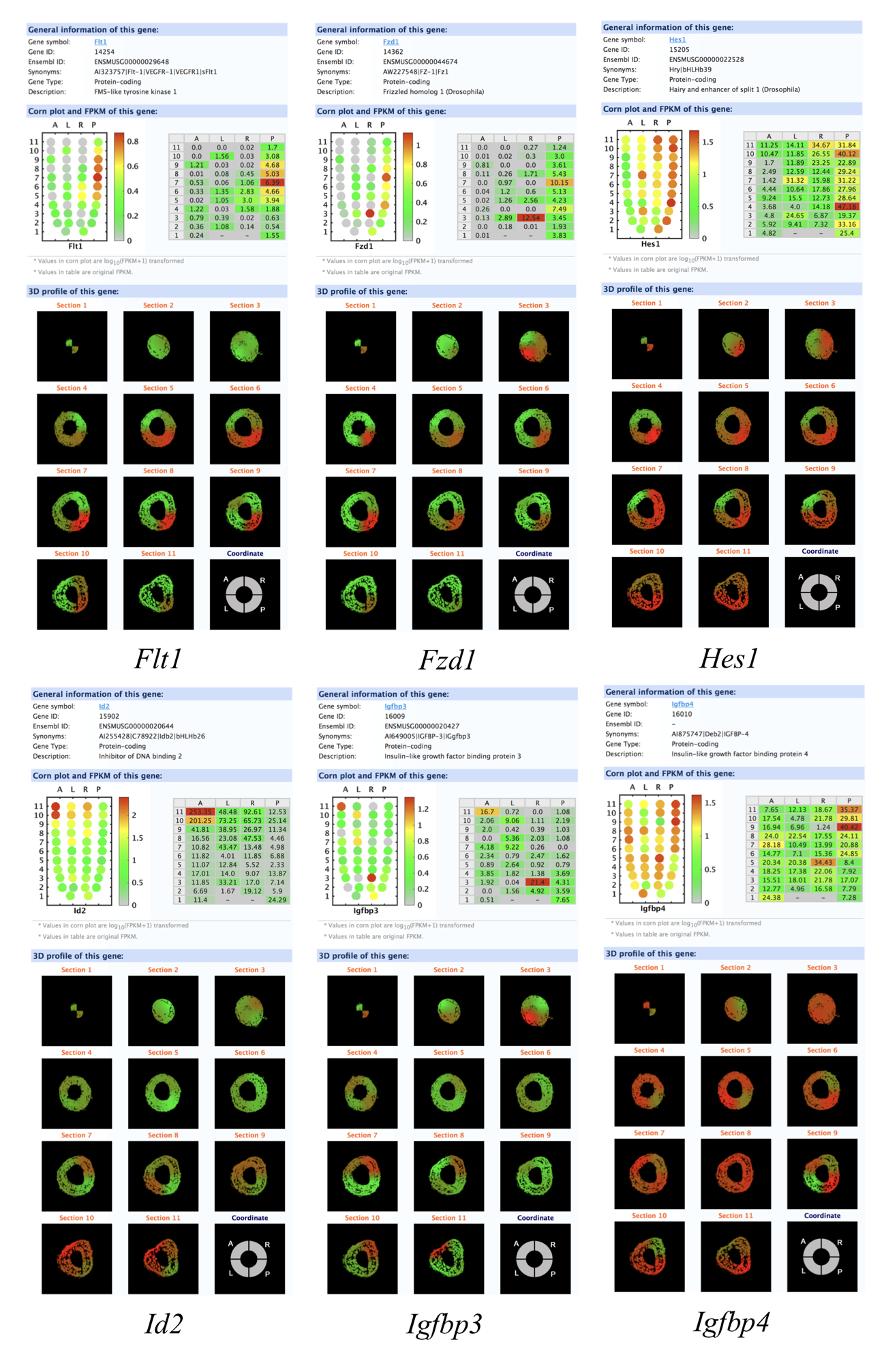


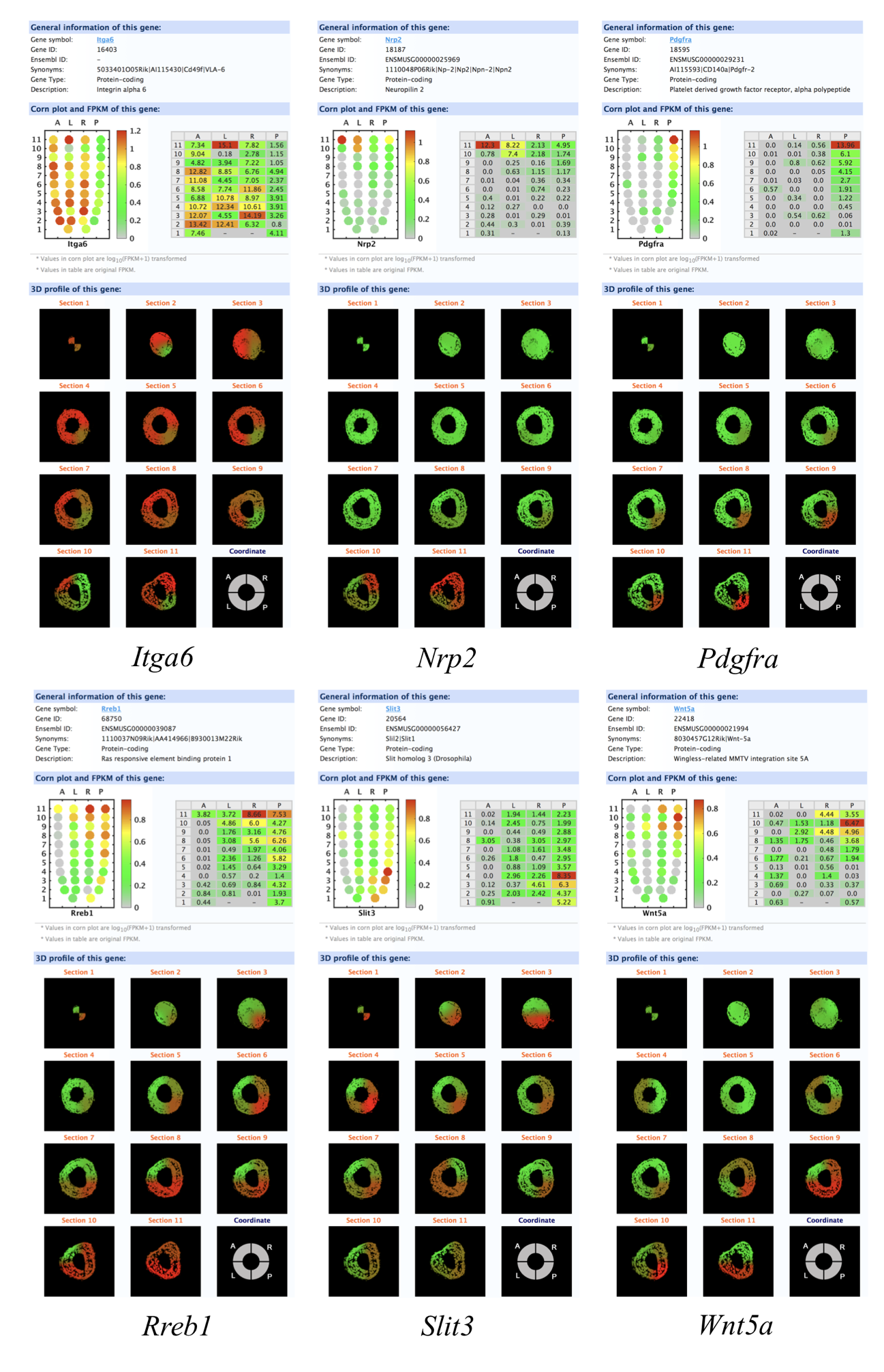


**Supplementary Figure S4.** Corn plots, FPKM, and 3D profiles of the 18 genes that achieved 100% success rate by iTranscriptome. The panels show iTranscriptome (<http://www.picb.ac.cn/hanlab/itranscriptome>) patterns for the 18 genes (*Acvr1*, *Cited2*, *Coro1b*, *Dll1*, *Enpp2*, *Fgfrl1*, *Flt1*, *Fzd1*, *Hes1*, *Id2*, *Igfbp3*, *Igfbp4*, *Itga6*, *Nrp2*, *Pdgfra*, *Rreb1*, *Slit3*, and *Wnt5a*) that achieved 100% success rate.

**Supplementary Figure S5.** SPRESSO analysis for the 18 gene expression data that produced 100% success rate (see Table 2) with noise. The points and the error bars show the mean values and the standard errors, respectively, of 10 replicates of random Gaussian noise experiments at 1-10% noise levels. As a result, for 1-5% noise levels, SPRESSO reproduced success rates as high as 80.3% or more, however, as the noise level increases the success rate gradually decreases to 65.8% at 10% noise level. Total variances were low (0.11 or less) at 1-5% noise levels, but increased to 0.12 at 10% noise level. These results indicate that our stochastic-SOM approach is robust to moderate noise levels (<5%) in the data.

**Supplementary Figure S6.** Evaluation of reconstructed structures of mid-gastrula mouse embryo. The mid-gastrula mouse embryo structure ((**a**), modified from Figure 2B of Ref. 27) and the 3D cubic model (**b**) are shown. The evaluation of the 3D reconstructed structure is performed by comparing the relative positions of all domains (D1-D4) in the cubic structure. The comparisons are performed such that: (i) D1 and D4 are positioned diagonally in the $xy$-direction, (ii) D2 and D3 are adjacent in the $z$-direction, and (iii) D*i* and D*j* are adjacent in the $xy$-direction, where $i\in\left\{ 1,4 \right\}$ and $j\in\{2,3\}$.

**Supplementary Figure S7.** Finding the nearest point to the line segment for visualization of the reconstructed model. Let $A$ and $B$ be the centroid vector of the unit to which sample $j$ belongs and the centroid vector of unit $k$ of the output layer of SOM, respectively. Point $X$ is the nearest point on line $AB$ from sample position $P.$ Distance $\left\| \boldsymbol{x} \right\|$ of line segment $AX$ can be calculated by $(\boldsymbol{b}\cdot\boldsymbol{p})/\left\| \boldsymbol{b} \right\|$, where $\boldsymbol{x}=\vec{AX}$, $\boldsymbol{b}=\vec{AB}$, $\boldsymbol{p}=\vec{AP}$, and $\left\| \cdot\right\|$ indicates the norm of the vector.

**Supplementary Figure S8.** Relationship between centroid coordinates of an output unit and a sample position. The coordinates of sample $j$ belonging to output unit $i$ are defined by $\left( x_{j}, y_{j}, z_{j} \right)=\left( x_{i}+d_{x}, y_{i}+d_{y}, z_{i}+d_{z} \right)$.

**Supplementary Tables**

**Supplementary Table S1.** Combinations of three GOs showing 96% or higher success rates.

| Combination of three GOs | Success rate | Total variance |
| --- | --- | --- |
| {GO:0060412, GO:0034707, GO:0044117} | 0.97 | 0.119 |
| {GO:0060412, GO:0034707, GO:0044130} | 0.97 | 0.119 |
| {GO:0060412, GO:0034707, GO:0044146} | 0.97 | 0.119 |
| {GO:0060412, GO:0002830, GO:1905456} | 0.96 | 0.125 |
| {GO:0060412, GO:0045630, GO:1905456} | 0.96 | 0.125 |

**Supplementary Table S2.** Combinations of four GOs showing 98% success rate.

| Combination of four GOs | Success rate | Total variance |
| --- | --- | --- |
| {GO:0060412, GO:0031994,  GO:0005021, GO:2000392} | 0.98 | 0.107 |
| {GO:0060412, GO:0031994,  GO:0005021, GO:2000394} | 0.98 | 0.107 |

**Supplementary Table S3.** Combinations of five GOs showing 99% success rate.

| Combination of five GOs | Success rate | Total variance |
| --- | --- | --- |
| {GO:0060412, GO:0005021, GO:2000392, GO:0031994, GO:0070986} | 0.99 | 0.105 |
| {GO:0060412, GO:0005021, GO:2000394, GO:0031994, GO:0070986} | 0.99 | 0.105 |

**Supplementary Table S4.** Combinations of six GOs showing 99% success rate.

| Combination of six GOs | Success rate | Total variance |
| --- | --- | --- |
| {GO:0060412, GO:0005021, GO:0031994, GO:2000392, GO:0070986, GO:2000394} | 0.99 | 0.105 |
| {GO:0060412, GO:0005021, GO:1905564, GO:1905456, GO:0072079, GO:0030169} | 0.99 | 0.121 |

**Supplementary Table S5.** 20 genes contained in the five GOs showing 99% or higher success rates.

| Gene | Official full name |
| --- | --- |
| *Acvr1* | activin A receptor, type 1 |
| *Arl13b* | ADP-ribosylation factor-like 13B |
| *Cited2* | Cbp/p300-interacting transactivator, with Glu/Asp-rich carboxy-terminal domain, 2 |
| *Coro1b* | coronin, actin binding protein 1B |
| *Dll1* | delta like canonical Notch ligand 1 |
| *Enpp2* | ectonucleotide pyrophosphatase/phosphodiesterase 2 |
| *Fgfrl1* | fibroblast growth factor receptor-like 1 |
| *Flt1* | FMS-like tyrosine kinase 1 |
| *Fzd1* | frizzled class receptor 1 |
| *Hes1* | hes family bHLH transcription factor 1 |
| *Id2* | inhibitor of DNA binding 2 |
| *Igfbp3* | insulin-like growth factor binding protein 3 |
| *Igfbp4* | insulin-like growth factor binding protein 4 |
| *Itga6* | integrin alpha 6 |
| *Nrp2* | neuropilin 2 |
| *Pdgfra* | platelet derived growth factor receptor, alpha polypeptide |
| *Rreb1* | ras responsive element binding protein 1 |
| *Slit3* | slit guidance ligand 3 |
| *Smad7* | SMAD family member 7 |
| *Wnt5a* | wingless-type MMTV integration site family, member 5A |

**Supplementary Table S6.** Pairs of genes showing 99% or higher success rates when removed.

| Pair of genes | Success rate | Total variance |
| --- | --- | --- |
| {*Arl13b*, *Smad7*} | 1.00 | 0.104 |
| {*Arl13b*, *Itga6*} | 0.99 | 0.105 |

**Supplementary Table S7.** Success rates and total variances when each gene is removed singularly from the 18 genes with 100% success rate.

| Removed gene | Success rate | Total variance |
| --- | --- | --- |
| *Coro1b* | 0.97 | 0.105 |
| *Dll1* | 0.97 | 0.106 |
| *Itga6* | 0.96 | 0.107 |
| *Igfbp3* | 0.95 | 0.103 |
| *Igfbp4* | 0.95 | 0.105 |
| *Cited2* | 0.95 | 0.110 |
| *Nrp2* | 0.91 | 0.107 |
| *Pdgfra* | 0.91 | 0.108 |
| *Fgfrl1* | 0.90 | 0.101 |
| *Slit3* | 0.89 | 0.105 |
| *Fzd1* | 0.89 | 0.109 |
| *Flt1* | 0.88 | 0.109 |
| *Hes1* | 0.88 | 0.109 |
| *Wnt5a* | 0.82 | 0.109 |
| *Rreb1* | 0.77 | 0.115 |
| *Enpp2* | 0.71 | 0.107 |
| *Acvr1* | 0.64 | 0.109 |
| *Id2* | 0.37 | 0.111 |

**Supplementary Table S8.** Success rates and total variances when gene *Id2* is removed in pairs with each of the 17 genes.

| Removed genes | Success rate | Total variance |
| --- | --- | --- |
| {*Id2*, *Igfbp4*} | 0.62 | 0.109 |
| {*Id2*, *Dll1*} | 0.48 | 0.107 |
| {*Id2*, *Slit3*} | 0.45 | 0.112 |
| {*Id2*, *Pdgfra*} | 0.44 | 0.108 |
| {*Id2*, *Igfbp3*} | 0.38 | 0.111 |
| {*Id2*, *Fgfrl1*} | 0.33 | 0.109 |
| {*Id2*, *Itga6*} | 0.33 | 0.111 |
| {*Id2*, *Flt1*} | 0.33 | 0.116 |
| {*Id2*, *Coro1b*} | 0.29 | 0.114 |
| {*Id2*, *Wnt5a*} | 0.27 | 0.112 |
| {*Id2*, *Cited2*} | 0.23 | 0.113 |
| {*Id2*, *Rreb1*} | 0.23 | 0.121 |
| {*Id2*, *Acvr1*} | 0.21 | 0.117 |
| {*Id2*, *Enpp2*} | 0.19 | 0.113 |
| {*Id2*, *Hes1*} | 0.15 | 0.114 |
| {*Id2*, *Fzd1*} | 0.11 | 0.124 |
| {*Id2*, *Nrp2*} | 0.07 | 0.123 |
